# A Review on the Terpenes from Genus *Vitex*

**DOI:** 10.3390/molecules21091179

**Published:** 2016-09-06

**Authors:** Jin-Long Yao, Shi-Ming Fang, Rui Liu, Mahmood Brobbey Oppong, Er-Wei Liu, Guan-Wei Fan, Han Zhang

**Affiliations:** 1 Tianjin State Key Laboratory of Modern Chinese Medicine, Tianjin University of Traditional Chinese Medicine, 312 Anshanxi Road, Nankai District, Tianjin 300193, China; yaojinlong1992@163.com (J.-L.Y.); fang_shiming@163.com (S.-M.F.); lr_8000@163.com (R.L.); mahmood.obrobbey@gmail.com (M.B.O.); liuwei628@sohu.com (E.-W.L.); 2 Key Laboratory of Formula of Traditional Chinese Medicine of Ministry of Education, Tianjin University of Traditional Chinese Medicine, 312 Anshanxi Road, Nankai District, Tianjin 300193, China; 3 School of Chinese Materia Medica, Tianjin University of Traditional Chinese Medicine, 312 Anshanxi Road, Nankai District, Tianjin 300193, China

**Keywords:** *Vitex* genus, chemical constituents, terpenes, pharmacological effects

## Abstract

The genus *Vitex,* which belongs to the *Verbenaceae* family, includes approximately 250 species. Some species of the genus *Vitex* have traditionally been used for the treatment of headaches, ophthalmodynia, coughs, asthma, premenopausal syndrome, etc. Chemical investigations indicate that the characteristic constituents of the genus *Vitex* are terpenes, and 210 of these compounds, including monoterpenoids, sesquiterpenoids, diterpenoids and triterpenoids, have been obtained from 12 species. Pharmacological studies had shown that these terpenes possess anti-inflammatory, antitumor, antibacterial, antioxidant activities, and so on. In this paper, the identity of these terpenes and their pharmacological effects are reviewed, which can provide references for further research regarding the chemistry and utilization of the *Vitex* species.

## 1. Introduction

The genus *Vitex* is one of the largest genus in the *Verbenaceae* family, with approximately 250 species. It is widely distributed, but mainly found in the tropical areas with a few in subtropical regions. The plants are mostly shrubs or arbors [1]. Many species in the *Vitex* genus have significant medicinal effects. The fruits of *Vitex trifolia* L. var. *simplicifolia* Cham. and *Vitex trifolia* L. are named Manjingzi in the 2015 edition of Chinese Pharmacopoeia. Manjingzi is a traditional Chinese medicine with wind-heat-dispersing action used in treating headaches, migraines and ophthalmodynia. The leaves of *V. negundo* var*. cannabifolia* have been used in China for the treatment of coughs, phlegm, and asthma [2]. Various parts of *V. negundo*, including the leaves, roots and seeds, have been locally used as traditional folk medicines since antiquity, particularly in China. It is commonly used for its analgesic, anti-inflammatory, anti-rheumatism, and insecticidal effects [3]. Many other species of the genus also have been explored and researched. These include *V. agnus-castus*, *V. limonifolia*, *V. altissima*, *V. rotundifolia*, *V. peduncularis*, *V. negundo* var*. cannabifolia*, *V. vestita*, *V.* *rehmannii*, etc.

Different types of secondary metabolites e.g., terpenes, flavonoids, lignans, phenolic acids, anthraquinones, etc., are present in species in this genus [4]. Terpenes are one of the major secondary metabolites, with different types including monoterpenoids, sesquiterpenoids, diterpenoids, and triterpenoids being isolated and characterized from the genus. Pharmacological studies have shown that terpenes have anti-inflammatory, antitumor, antibacterial, antioxidant, hepatoprotective activities and so on. The goal of this review is to provide an overview of the chemical identities and the pharmacological effects of the terpenes isolated from species in the genus, which can serve as reference for further research and utilization of the *Vitex* species.

## 2. Chemical Constituents

**Figure 1:**
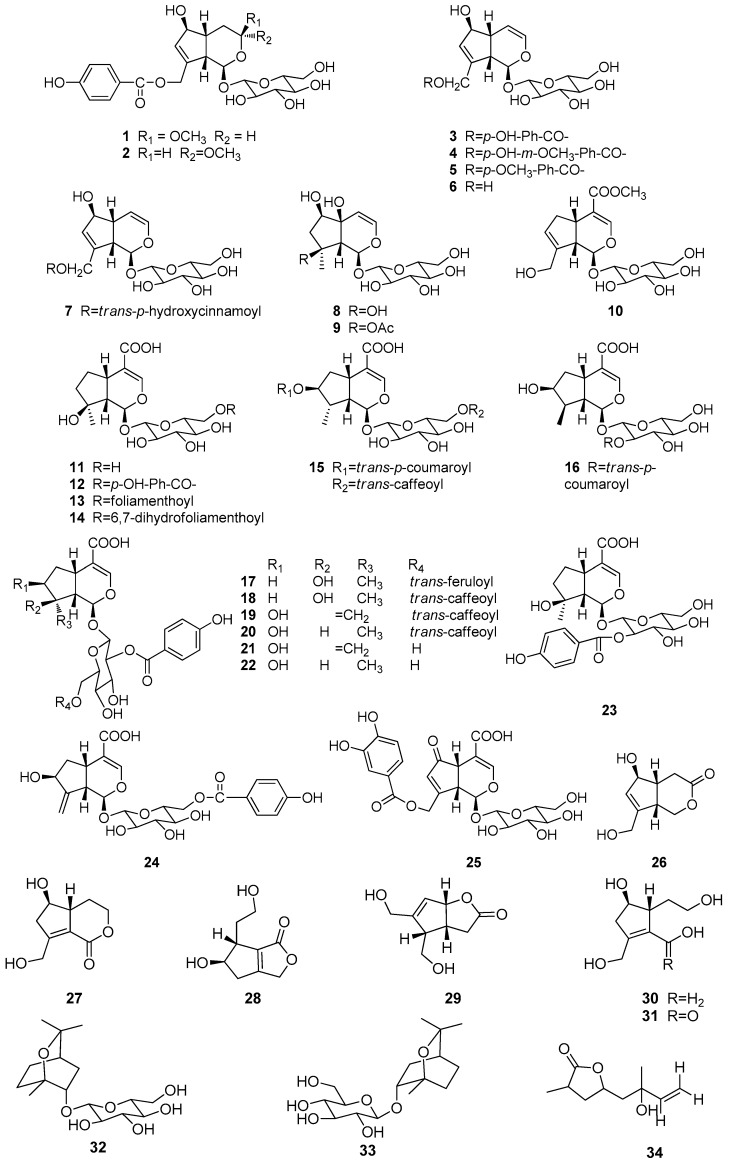
Structures of monoterpenoids **1**–**34** isolated from plants of *Vitex* L.

**Figure 2:**
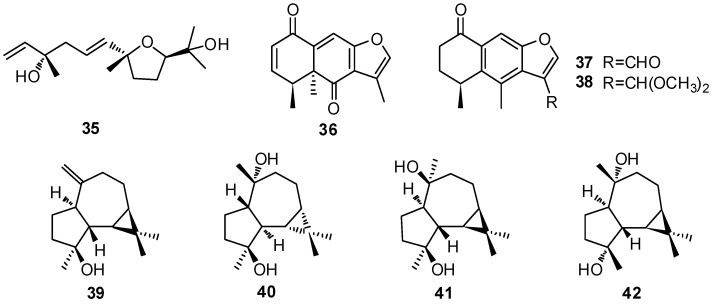
Structures of sesquiterpenoids **35**–**42** isolated from plants of *Vitex* L.

**Figure 3:**
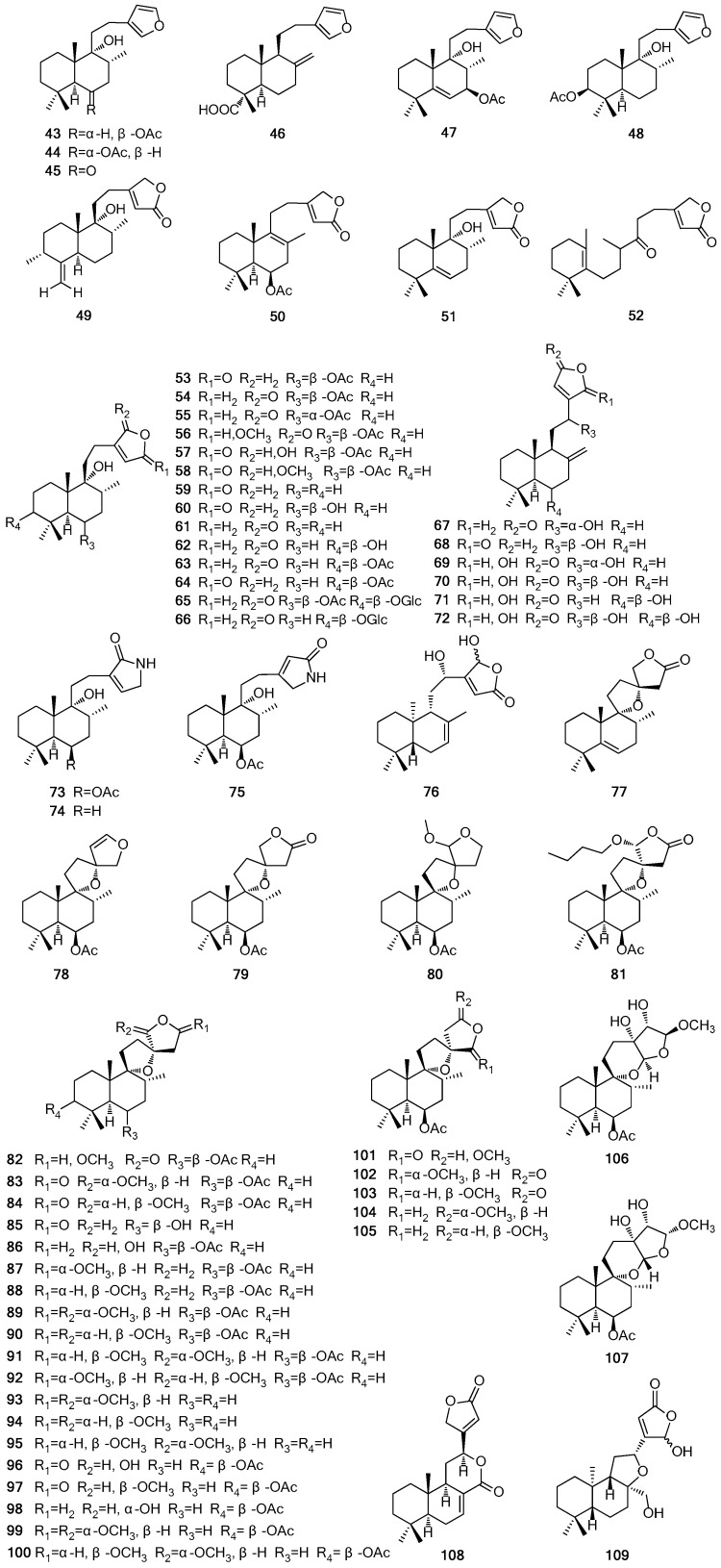

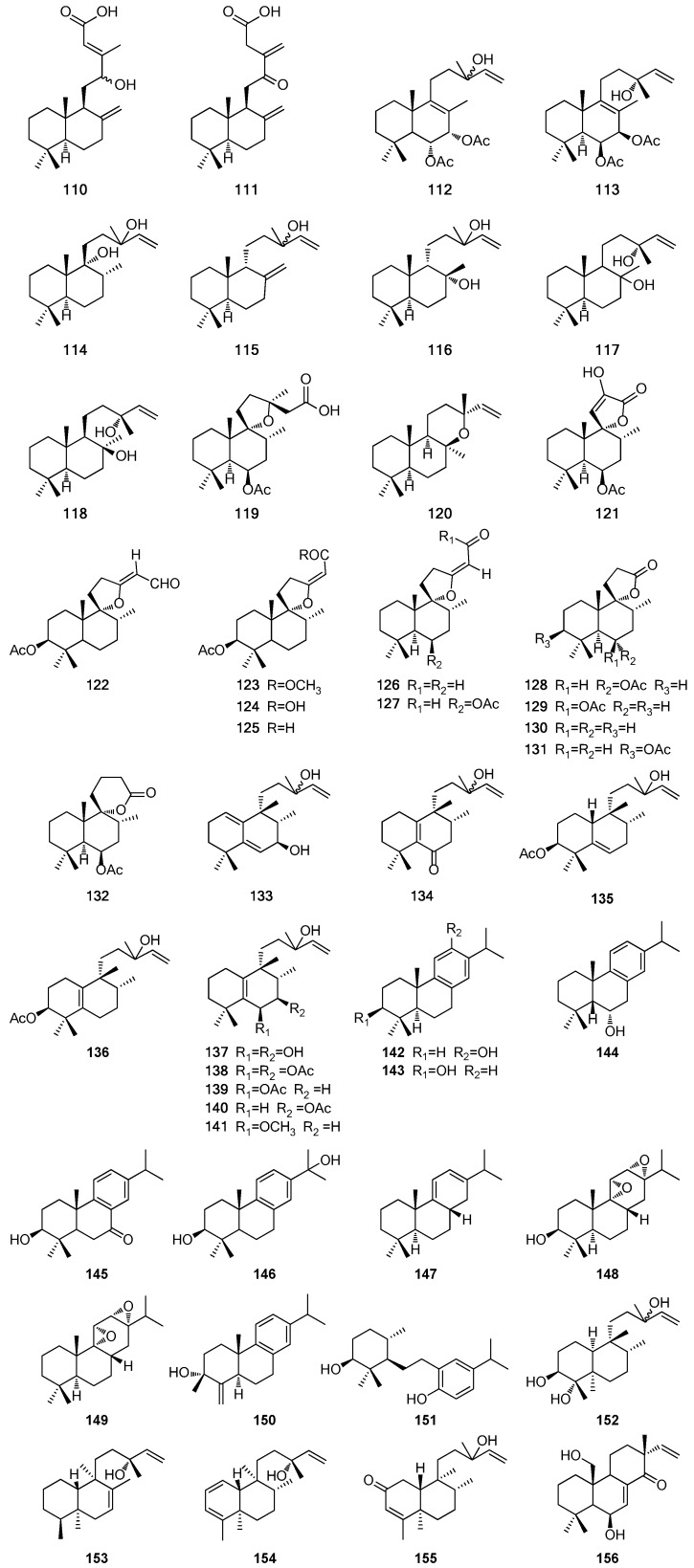
Structures of diterpenoids **43**–**156** isolated from *Vitex* L.

**Figure 4:**
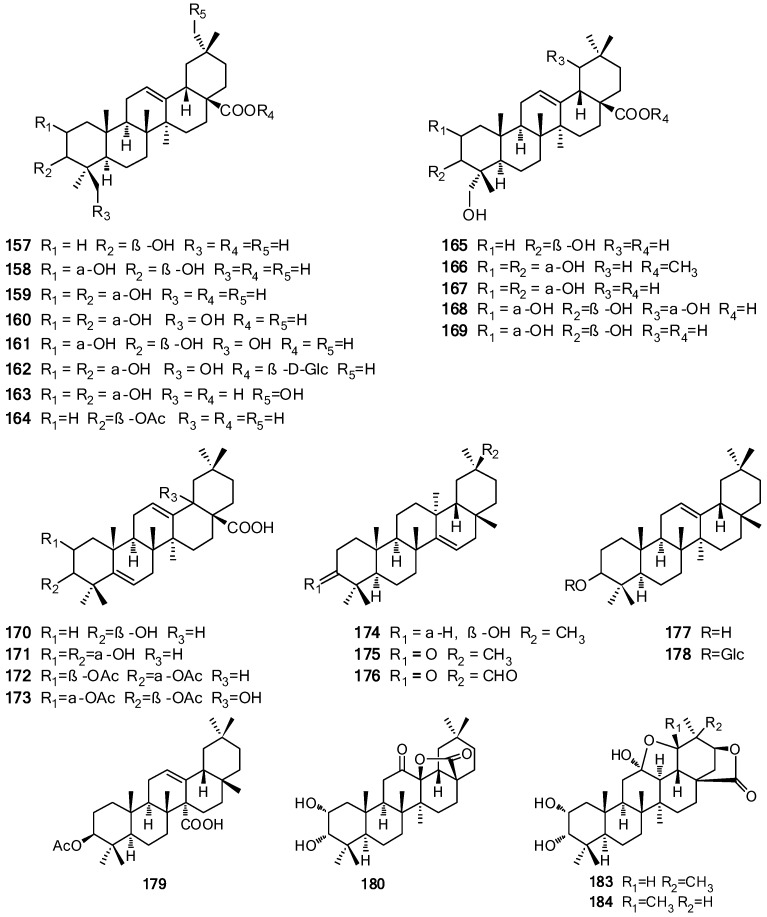

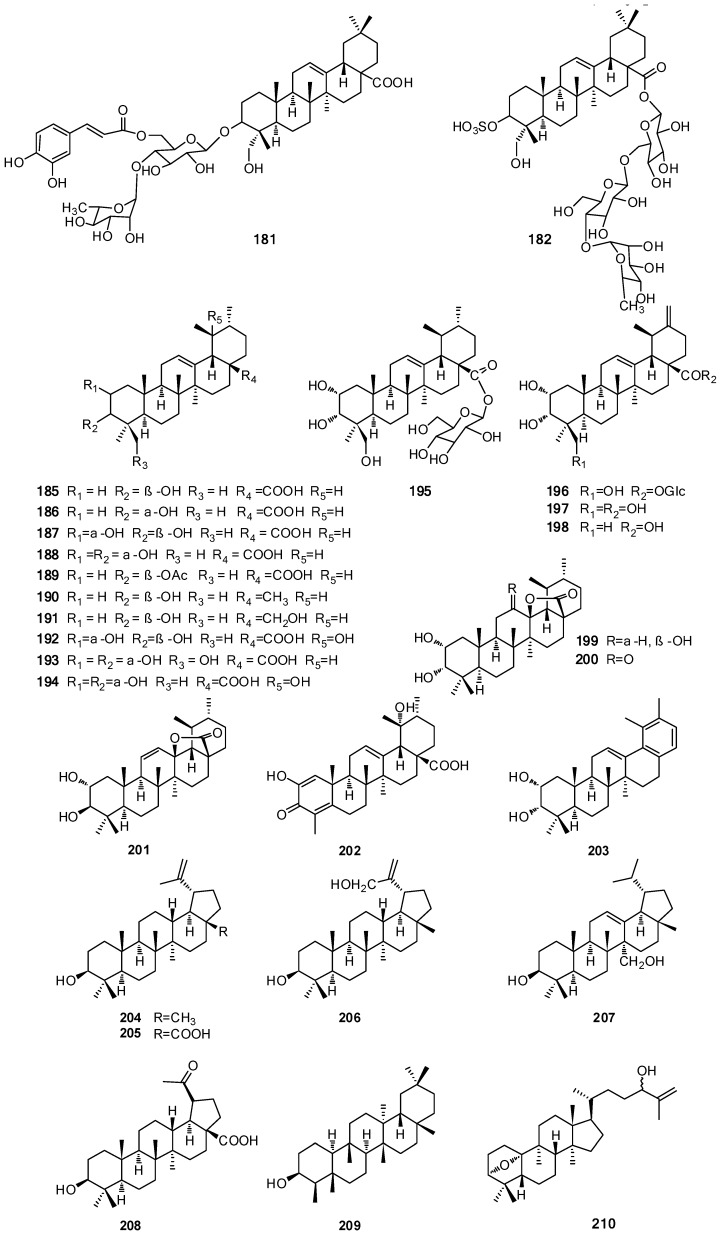
Structures of triterpenoids **157**–**210** isolated from *Vitex* L.

So far, more than 200 terpenes have been obtained from the different parts of *Vitex* plants. Among these compounds, diterpenoids are the most dominant terpenes reported in the species. The names of terpenes, the corresponding plant sources and references from which they are derived are summarized in Table 1, Table 2, Table 3 and Table 4. Their structures are shown in Figure 1, Figure 2, Figure 3 and Figure 4.

### 2.1. Monoterpenoids and Sesquiterpenoids

#### 2.1.1. Monoterpenoids

The majority of the monoterpenoids (Table 1, Figure 1) of the *Vitex* genus are iridoids and their corresponding glucosides (compounds **1**–**31**). Beside the iridoids, two cineole-type monoterpenoid glucosides **32**, **33** were obtained from the fruits of *Vitex rotundifolia* [5]. Moreover, Wu et al. [6] isolated an acyclic monoterpenoid vitexoid **34** from the fruits of *Vitex trifolia*.

#### 2.1.2. Sesquiterpenoids

Apart from the monoterpenoids, only eight sesquiterpenoids **35**–**42** were found in the *Vitex* plants (Table 2, Figure 2). Among them, negunfurol (**35**) is a new sesquiterpenoid from *V. negundo* containing a furan ring [28]. Tiwari et al. [29] isolated three sesquiterpenoids **36**–**38** with furanoeremophilane skeletons from the stems of *V. negundo*. Meanwhile, aromadendrane-type sesquiterpenoids **39**–**42** have been obtained from *V. trifolia*, *V. agnus-castus* and *V. poligama* [17,18,30,31,32,33].

### 2.2. Diterpenoids

Diterpenoids are abundant in the *Vitex* plants. The labdane-type diterpenoids **43**–**120** form the majority of the characterized diterpenoids, with the few others being norlabdane-type (compounds **121**–**132**), halimane-type (compounds **133**–**141**), abietane-type (compounds **142**–**151**), clerodane-type (compounds **152**–**155**) and isopimarane-type (compound **156**). Commonly, diterpenoids of the genus exist in the form of aglycones, and only **65** and **66** are diterpenoid glucosides which are rare in *Vitex* genus [33,34]. Compounds **73**, **74** and **75** are found as diterpenoid alkaloids containing an α,β-unsaturated-γ-lactam moiety, and these structures are unique in the genus [18,35,36]. Zheng et al. [37] isolated a 9,10-*seco* abietane diterpenoid negundoin F (**151**) and an isopimarane-type diterpenoid negundoin G (**156**) from an ethanolic extract of the seeds of *V. negundo.* The names of diterpenoids and their structures are listed and shown in Table 3 and Figure 3, respectively.

### 2.3. Triterpenoids

The triterpenoids isolated from the genus are mainly pentacyclic triterpenoids, consisting of oleanane-type (compounds **157**–**183**), ursane-type (compounds **184**–**201**), norursane-type (compounds **202**–**203**), lupane-type (compounds **204**–**208**) and friedelane-type (**209**). Only a few (compounds **162**, **178**, **181**, **182**, **195**, **196**) are triterpenoid glycosides [67,68]. Among them, cannabifolins A (**184**) and B (**183**) are the first examples of 12,19-epoxyursane- and oleanane-type triterpenoids and are rare natural pentacyclic triterpenoids with *cis*-fused C/D rings [69]. Tetracyclic triterpenoids like the 9-*epi*-cucurbitane-type **210** also has been isolated [43]. The names of these triterpenoids and their structures are listed in Table 4 and shown in Figure 4, respectively.

## 3. Pharmacological Effects

Terpenes isolated from *Vitex* plants have been evaluated for their anti-inflammatory, anti-tumor, antibacterial, antioxidant and other pharmacological effects, which provide potential explanations for their use in the treatment of various diseases in folk medicine. It was proved that terpenes were the principal active constituents for the aforementioned effects. A detailed summary of their pharmacological studies is given below.

### 3.1. Anti-Inflammatory Activity

Many plants from *Vitex* genus have been used for the treatment of inflammatory diseases. And pharmacological studies have also shown that some terpenes isolated from the genus have significant anti-inflammatory effects. Agnuside (**3**) exerted significant anti-inflammatory activity using carrageenan-, histamine- and dextran-induced acute inflammation models in rats. The inhibitory effect seemed independent of activation of the pituitary-adrenal axis because the inhibition effects against carrageenan-induced oedema in normal and adrenalectomized rats after oral administration of agnuside (**3**) were highly comparable. Furthermore, oral administration of agnuside (**3**) to arthritic rats can decrease the levels of intracellular interleukin-17 (IL-17) in lymphocytes with values of 12.17% and 11.04% at doses of 6.12 and 12.5 mg/kg, compared with non-agnuside-fed control groups 19.71% [20]. Twenty-four different compounds were isolated from *V. rotundifolia* by Lee et al., and their anti-inflammatory activities were tested by the Griess method. The results revealed that five diterpenoids (compounds **57**, **61**, **106**, **141**, **138**) significantly inhibited nitric oxide (NO) production in lipopolysaccharide (LPS)-stimulated RAW 264.7 cells with the IC_50_ values of 11.3, 16.4, 17.2, 22.2 and 24.5 μM respectively, while the positive control aminoguanidine being 16.6 μM [45]. Li et al. [69] isolated fourteen triterpenoids from *V. negundo* var. *cannabifolia*, of which five compounds (**192**, **198**, **159**, **199**, and **160**) demonstrated moderate inhibitory effects on NO production, with IC_50_ values of 24.9 ± 4.6, 26.1 ± 3.6, 27.7 ± 3.3, 34.0 ± 4.1, 40.5 ± 4.9 μM, respectively. Zheng et al. [37] have isolated nine diterpenoids (compounds **97**, **98**, **123**–**125**, **131**, **145**, **151**, **156**) from the seeds of *V. negundo*. Among these, negundoin C (**125**) and negundoin E (**98**) showed the most significant inhibitory effects on NO production using LPS-stimulated RAW 264.7 cells, with IC_50_ values of 0.12 and 0.23 μM, respectively, compared with the positive control indomethacin at 45.51 μM. Additionally, the authors demonstrated the protein expressions of cyclooxygenase-2 (COX-2) and inducible nitric oxide synthetase (iNOS) with western blot analysis to describe the possible mechanism of their anti-inflammatory activity, and it was an interesting finding that the level of COX-2 protein and iNOS protein were decreased by **98** and **125**.

### 3.2. Anti-Tumor Activity

It is also worth mentioning that some terpenes of the *Vitex* genus possess significant anti-tumor activities against several cancer cell lines. Wu et al. [6] isolated ten diterpenoids **34**, **48**, **53**, **54**, **79**, **80**, **137**–**140**, including three new compounds **34**, **48**, **137**, from *V. trifolia* L. All compounds were tested for their inhibitory effects on HeLa cell proliferation with the MTT assay, and their IC_50_ values ranged from 4.9 ± 0.5 to 28.7 ± 1.3 μM. Furthermore, vitetrifolin I (**137**) exhibited significant inhibition effect with an IC_50_ value of 4.9 ± 0.5 μM, and induced cell cycle G_0_/G_1_ phase arrest and apoptosis of HeLa cells. Six terpenes **35**, **37**, **122**, **188**, **202**, **203** were isolated from *V. negundo* and evaluated for their cytotoxicities against four cancer cell lines using the MTT method. The results revealed that negunfurol (**35**) was the most active compound against HL-60, with an IC_50_ value of 0.94 ± 0.26 μg/mL and negundonorin A (**202**) was highly cytotoxic to ZR-75-30 cells with an IC_50_ value of 0.56 ± 0.19 μg/mL [28]. Mahesh et al. [35] isolated six diterpenoids **43**, **53**, **74**, **75**, **120**, **138**, including a new diterpenoid alkaloid **74**, from *V. agnus-castus*. All compounds were evaluated for their cytotoxicities against the K562 cell line. The IC_50_ values ranged from 0.70 to 6.72 μg/mL, and compound **74** was the most cytotoxic, with an IC_50_ value of 0.70 μg/mL, compared with the positive control cisplatin at 1.10 μg/mL. Corlay et al. [55] isolated nine labdane-type diterpenoids **67**–**72**, **109**–**111** from *V. vestita*. All the diterpenoids except vitexolin A (**110**) were cytotoxic against the HCT-116 and MRC-5 cancer cell lines to some extent.

### 3.3. Antibacterial and Antifungal Activities

According to references [44,55,59,84], some terpenes in the *Vitex* genus possess significant antibacterial and antifungal activities. Vitexilactone C (**49**) showed weak antibacterial activity against *Bacillus subtilis*, *Escherichia coli* and *Micrococcus* *tetragenus* at the same minimum inhibitory concentration (MIC) value of 500 μg/mL [44]. The diterpenoid vitexolide A (**69**) isolated from *V. vestita* showed the most potent antibacterial activity against 46 Gram-positive strains compared with other diterpenoids **67**, **68**, **70**, **109**, **111**. The MIC values ranged from 6 to 96 μM [55]. Epifriedelinol (**209**) is a pentacyclic triterpenoid isolated from *Vitex peduncularis* by bioassay guided separation. Its antibacterial activity was tested against 12 strains of Gram positive and Gram negative bacteria. The MIC values were in the range of 6.25–50 μg/mL. The minimum bactericidal concentration (MBC) values were in the range of 12.5–100 μg/mL [84]. Additionally, negundol (**96**), a labdane-type diterpeoid isolated from the seeds of *V. negundo* exhibited antifungal activity against *Candida albicans* (MIC_80_: 64 μg/mL), *Cryptococcus* *neoformans* (MIC_80_: 16 μg/mL) and *Trichophyton rubrum* (MIC_80_: 32 μg/mL) [59].

### 3.4. Antioxidant Activity

Results from different studies have demonstrated that many terpenes in the *Vitex* genus have significant antioxidant activites. Sridhar et al. [13] isolated six new acylated iridoid glucosides (compounds **17**–**22**) from *V. altissima*, and each compound was tested for its superoxide radical-scavenging activity using the McCord and Fridovich method and 1-diphenyl-2-picrylhydrazyl (DPPH) radical-scavenging effect with the Lamaison method. The results showed three compounds **18**–**20** exhibiting significant antioxidant activity by both methods. Tiwari et al. [21] isolated iridoid glucosides agnuside (**3**), negundoside (**23**) and 6′-*O*-*p*-hydroxybenzoyl mussaenosidic acid (**12**) from *V. trifolia*. Compounds **3**, **23** and **12** showed DPPH radical scavenging avtivities with IC_50_ values of 9.81, 9.96 and 10.31 μg, respectively, and also effectively inhibited NO radical at IC_50_ values of 12.90, 16.25 and 13.51 μg. Ferruginol (**142**), an abietane-type diterpenoid isolated from *V. rotundifolia* showed higher antioxidant activity than 3-*tert*-butyl-4-hydroxyansiole (BHA) using the ferric thiocyanate method. Futhermore, it has stronger DPPH radical scavenging effect equivalent to half that of l-cysteine [58].

### 3.5. Other Pharmacological Activities

Additionally, some of the terpenes also have analgesic, endocrinological, anti-hyperglycemic, antifeedant effects, etc. Okuyama et al. [15] verified the analgesic effect of two iridoids agnuside (**3**) and 10-*O*-vanilloylaucubin (**4**) by the acetic acid induced writhing test in mice. At a dose of 50 mg/kg compounds **3** and **4** exerted analgesic effects of 56% (*p* < 0.001) and 20% (*p* < 0.05), respectively. Extracts of *V. agnus-castus* have been used for amelioration of premenopausal syndrome, especially mastodynia, which were most likely caused by hypersecretion of prolactin. The proposed mechanism of action was due to dopaminergic and estrogenic principle. The mixture of clerodane-type diterpenoids (BNO-diterpenoids), isolated from 70% ethanolic extract of *V. agnus-castus*, showed the highest dopaminergic activity by reducing cyclic AMP (cAMP) formation and prolactin secretion [41]. Sundarama et al. [26] obtained the iridoid glucoside **23** from leaves of *V. negundo*, which could reduce the levels of blood glucose and glycoproteins, and increase the level of plasma insulin in streptozotocin diabetic rats. Compound **23** also showed anti-hyperlipidemic activity [27]. Additionally, hepatoprotective activity of some terpenes (compounds **23**, **164**) from genus *Vitex* plants was discovered by Indian scholars [24,75,85]. Ursolic acid (**185**) and betulinic acid (**205**) showed antifeedant activity against the larvae of *Achoea janata* [81].

## 4. Conclusions

In this review, we summarize the research progress on terpenes of the genus *Vitex* and their pharmacology. These findings indicate that this genus is a valuable source of bioactive molecules. Phytochemical and pharmacological studies of the compounds isolated from the genus *Vitex* have attracted more attention in recent years. Terpenes, including monoterpenoids, sesquiterpenoids, diterpenoids and triterpenoids were identified as the main chemical constituents of this genus. From the literature, there are approximately 250 species in the genus [1], but studies on terpenes had been done to some extent on only 12 species [9,10,11,12,13,14,15,16,17,18,19,20,21,30,55,56,62]. Considering the many bioactive terpenes isolated from the plants in this genus, further investigations on terpenes and their pharmacological effects of the other species are very necessary. In the pharmacology domain, most of the isolated terpenes have been evaluated for various activities in vitro without being further tested in vivo. Thus the promising pharmacological activities should be confirmed by in vivo assay using diverse rat models to prove them. In addition, taking into account their therapeutic efficiency, validating the relationships between chemical constituents, pharmacological effects and traditional uses of plants in this genus is still remains a fundamental task, and should be paid more attention to.

## Figures and Tables

**Table 1:** Monoterpenoids **1**–**34** isolated from plants of *Vitex* L.

No.	Compound Name	Source	Reference
**1**	Nishindaside	a, h	[7,8,9]
**2**	Isonishindaside	h	[8]
**3**	Agnuside	a–h	[9,10,11,12,13,14,15,16,17,18,19,20,21]
**4**	10-*O*-Vanilloyl aucubin	d, e, h	[9,14,15,16]
**5**	Limoniside	g	[10]
**6**	Aucubin	f	[11,18]
**7**	Eurostoside	d	[12]
**8**	Harpagide	f	[22]
**9**	8-*O*-Acetylharpagide	f	[22]
**10**	Geniposide	h	[9]
**11**	Mussaenosidic acid	f	[11]
**12**	6′-*O*-*p*-Hydroxybenzoylmussaenosidic acid	a, b, f	[11,21,23]
**13**	Agnucastoside A	f	[11]
**14**	Agnucastoside B	f	[11]
**15**	Agnucastoside C	f	[11]
**16**	2′-*O*-*trans*-*p*-Coumaroylloganic acid	a	[23]
**17**	6′-*O*-*trans*-Feruloylnegundoside	c	[13]
**18**	6′-*O*-*trans*-Caffeoylnegundoside	c	[13]
**19**	2′-*O*-*p*-Hydroxybenzoyl-6′-*O*-trans-caffeoylgardoside	c	[13]
**20**	2′-*O*-*p*-Hydroxybenzoyl-6′-*O*-trans-caffeoyl-8-epiloganic acid	c	[13]
**21**	2′-*O*-*p*-Hydroxybenzoyl gardoside	c	[13]
**22**	2′-*O*-*p*-Hydroxybenzoyl-8-epiloganic acid	c	[13]
**23**	Negundoside	a, c	[7,13,23,24,25,26,27]
**24**	6′-*O*-*p*-Hydroxybenzoyl-gardoside	a	[23]
**25**	1,4a,5,7a-Tetrahydro-1-β-d-glucosyl-7-(3′,4′-dihydroxybenzoyloxymethyl)-5-ketocyclopenta[*c*]pyran-4-carboxylic acid	a	[7]
**26**	Iridolactone	d	[14]
**27**	Viteoid II	d	[14]
**28**	Viteoid I	d	[14]
**29**	Pedicularis lactone	d	[14]	
**30**	Eucommiol	d	[14]	
**31**	1-Oxoeucommiol	d	[14]	
**32**	(1*S*,2*S*,4*R*)-2-*endo*-Hydroxy-1,8-cineole-β-d-glucopyranoside	d	[5]	
**33**	(1*R*,2*R*,4*S*)-2-*endo*-Hydroxy-1,8-cineole-β-d-glucopyranoside	d	[5]	
**34**	Vitexoid	b	[6]	

a: *Vitex negundo*. b: *V. trifolia*. c: *V. altissima*. d: *V. rotundifolia*. e: *V. peduncularis*. f: *V. agnus-castus*. g: *V. limonifolia*. h: *V. negundo* var. *cannabifolia* (syn.: *V. cannabifolia*).

**Table 2:** Sesquiterpenoids **35**–**42** isolated from plants of *Vitex* L.

No.	Compound Name	Source	Reference
**35**	Negunfurol	a	[28]
**36**	1,6-Dioxo-2(3),9(10)-dehydrofuranoeremophilane	a	[29]
**37**	4,6-Dimethyl-11-formyl-1-oxo-4*H*,2,3-dihydronaphthofuran	a	[28,29]
**38**	4,6-Dimethyl-11-dimethoxymethyl-1-oxo-4*H*,2,3-dihydronaphthofuran	a	[29]
**39**	Spathulenol	b, f, i	[17,18,30,31]
**40**	*ent*-4α,10β-Dihydroxyaromadendrane	b	[17]
**41**	4β,10β-Dihydroxyaromadendrane	f	[32]
**42**	4α,10α-Dihydroxyaromadendrane	f	[33]

a: *Vitex negundo*. b: *V. trifolia*. f: *V. agnus-castus*. i: *V. poligama*.

**Table 3:** Diterpenoids **43**–**156** isolated from plants of *Vitex* L.

Type	No.	Compound Name	Source	Reference
Labdane	**43**	Rotundifuran	b, d, f	[31,35,38,39,40,41,42]
	**44**	Vitetrifolin B	b, f	[31,39]
	**45**	Dihydrosolidagenone	b	[39]
	**46**	(+)-Polyalthic acid	a	[43]
	**47**	Vitetrifolin C	b, f	[31,39]
	**48**	Vitetrifolin H	b	[6,38]
	**49**	Vitexilactone C	h	[44]
	**50**	Vitextrifolin C	b	[38]
	**51**	Vitextrifolin D	b	[38]
	**52**	Vitextrifolin E	b	[38]
	**53**	Vitexilactone	b, d, f	[6,31,33,35,36,40,45,46,47,48,49,50]
	**54**	(*rel* 5*S*,6*R*,8*R*,9*R*,10*S*)-6-Acetoxy-9-hydroxy-13(14)-labden-16,15-olide	b,d	[6,48,50]
	**55**	(*rel* 5*S*,6*S*,8*R*,9*R*,10*S*)-6-Acetoxy-9-hydroxy-13(14)-labden-16,15-olide	d	[50]
	**56**	(*rel* 5*S*,6*R*,8*R*,9*R*,10*S*)-6-Acetoxy-9-hydroxy-15-methoxy-13(14)-labden-16,15-olide	d	[46,50]
	**57**	Viteagnusin I	b, d, f	[38,45,51]
	**58**	Viteagnusin H (methoxy-vitexilactone)	f	[32,52]
	**59**	9-Hydroxy-13(14)-labden-15,16-olide	b	[53]
	**60**	Deacetylvitexilactone	b	[38]
	**61**	Viterotulin A	d	[45]
	**62**	(*rel* 3*S*,5*S*,8*R*,9*R*,10*S*)-3,9-Dihydroxy-13(14)-labden-16,15-olide	d	[45]
	**63**	Viterotulin B	d	[45]
	**64**	Vitexilactone B	a, b	[38,54]
	**65**	Viteoside A	d	[34]
	**66**	Viteagnuside A	f	[33]
	**67**	Vitexolide E	j	[55]
	**68**	Vitexolide D	j	[55]
	**69**	Vitexolide A	j	[55]
	**70**	12-Epivitexolide A	j	[55]
	**71**	Vitexolide B	j	[55]
	**72**	Vitexolide C	j	[55]
	**73**	Vitexlactam A	d,f	[18,36]
	**74**	Vitexlactam B	f	[18,35]
	**75**	Vitexlactam C	f	[18,35]
	**76**	12*S*,16*S*/*R*-Dihydroxy-*ent*-labda-7,13-dien-15,16-olide	k	[56]
	**77**	Vitextrifolin G	b	[38]
	**78**	Prerotundifuran	d	[42]
	**79**	Previtexilactone	b, d	[6,38,47,48,49]
	**80**	6-Acetoxy-9,13,15,16-diepoxy-15-methoxylabdane	b	[6]
	**81**	Viteagnusin E	f	[57]
	**82**	(*rel* 5*S*,6*R*,8*R*,9*R*,10*S*,13*S*)-6-Acetoxy-9,13-epoxy-15-methoxylabdan-16,15-olide	d, f	[33,50]
	**83**	Viteagnusin I	f	[33]
	**84**	(*rel* 5*S*,6*R*,8*R*,9*R*,10*S*,13*S*,16*S*)-6-Acetoxy-9,13-epoxy-16-methoxy-labdan-15,16-olide	d, f	[33,50]
	**85**	Vitextrifolin F	b	[38]
	**86**	Nishindanol	a	[19]
	**87**	(*rel* 5*S*,6*R*,8*R*,9*R*,10*S*,13*S*,15*S*)-6-Acetoxy-9,13,15,16-diepoxy-15-methoxylabdane	d, f	[32,58]
	**88**	(*rel* 5*S*,6*R*,8*R*,9*R*,10*S*,13*S*,15*R*)-6-Acetoxy-9,13;15,16-diepoxy-15-methoxylabdane	d, f	[32,58]
	**89**	(*rel* 5*S*,6*R*,8*R*,9*R*,10*S*,13*S*,15*S*,16*R*)-6-Acetoxy-9,13,15,16-diepoxy-15,16-dimethoxylabdane	d	[58]
	**90**	(*rel* 5*S*,6*R*,8*R*,9*R*,10*S*,13*S*,15*R*,16*S*)-6-Acetoxy-9,13,15,16-diepoxy-15,16-dimethoxylabdane	d	[58]
	**91**	(*rel* 5*S*,6*R*,8*R*,9*R*,10*S*,13*S*,15*R*,16*R*)-6-Acetoxy-9,13,15,16-diepoxy-15,16-dimethoxylabdane	d	[58]
	**92**	(*rel* 5*S*,6*R*,8*R*,9*R*,10*S*,13*S*,15*S*,16*S*)-6-Acetoxy-9,13,15,16-diepoxy-15,16-dimethoxylabdane	d	[58]
	**93**	(*rel* 5*S*,8*R*,9*R*,10*S*,13*S*,15*S*,16*R*)-9,13;15,16-Diepoxy-15,16-dimethoxylabdane	d	[50]
	**94**	(*rel* 5*S*,8*R*,9*R*,10*S*,13*S*,15*R*,16*S*)-9,13;15,16-Diepoxy-15,16-dimethoxylabdane	d	[50]
	**95**	(*rel* 5*S*,8*R*,9*R*,10*S*,13*S*,15*R*,16*R*)-9,13;15,16-Diepoxy-15,16-dimethoxylabdane	d	[50]
	**96**	Negundol	a, b	[38,59]
	**97**	Negundoin D	a	[37]
	**98**	Negundoin E	a	[37]
	**99**	Vitextrifolin A	b	[38]
	**100**	Vitextrifolin B	b	[38]
	**101**	(*rel* 5*S*,6*R*,8*R*,9*R*,10*S*,13*R*)-6-Acetoxy-9,13-epoxy-15-methoxylabdan-16,15-olide	d,f	[33,50,57]
	**102**	(*rel* 5*S*,6*R*,8*R*,9*R*,10*S*,13*R*,16*S*)-6-Acetoxy-9,13-epoxy-16-methoxylabdan-15,16-olide	d,f	[33,50]
	**103**	Viteagnusin J	f	[33]
	**104**	(*rel* 5*S*,6*R*,8*R*,9*R*,10*S*,13*R*,15*R*)-6-Acetoxy-9,13,15,16-diepoxy-15-methoxylabdane	d, f	[32,58]
	**105**	(*rel* 5*S*,6*R*,8*R*,9*R*,10*S*,13*R*,15*S*)-6-Acetoxy-9,13,15,16-diepoxy-15-methoxylabdane	d, f	[32,58]
	**106**	Viteagnusin F	d, f	[32,45]
	**107**	Viteagnusin G	d, f	[32,45]
	**108**	Limonidilactone	g	[60]
	**109**	Acuminolide	j	[55]
	**110**	Vitexolin A	j	[55]
	**111**	Vitexolin B	j	[55]
	**112**	6α,7α-Diacetoxy-13-hydroxy-8(9),14-labdadiene	b	[53]
	**113**	6β,7β-Diacetoxy-13-hydroxylabda-8,14-diene	f	[40,41]
	**114**	Viteagnusin D	f	[57]
	**115**	Vitexifolin A	d	[61]
	**116**	Viteagnusin C	a, f	[33,54,57]
	**117**	8,13-Dihydroxy-14-labdene	f	[41]
	**118**	8-*epi*-Sclareol	a, f	[19,33,54,57]
	**119**	Vitrifolin B	d	[36]
	**120**	8-Epimanoyl oxide	f	[18,35,51]
Norlabdane	**121**	Vitrifolin A	l	[62]
	**122**	Negundoal	a	[28]
	**123**	Negundoin A	a	[37]
	**124**	Negundoin B	a	[37]
	**125**	Negundoin C	a	[37]
	**126**	9,13-Epoxy-16-norlabda-13*E*-en-15-al (norditerpene aldehyde 1)	b, d	[45,48]
	**127**	Norditerpene aldehyde 2	b	[48]
	**128**	Vitexifolin D	d	[61]
	**129**	Trisnor-γ-lactone	d	[61]
	**130**	Isoambreinolide	b, d	[53,61]
	**131**	Vitedoin B	a, d	[37,45,63]
	**132**	Vitexifolin E	d	[61]
Halimane	**133**	Vitetrifolin G	b	[64]
	**134**	13-Hydroxy-5(10),14-halimadien-6-one	b	[53]
	**135**	Viteagnusin A	f	[57]
	**136**	Viteagnusin B	f	[57]
	**137**	Vitetrifolin I	b	[6]
	**138**	Vitetrifolin D	a, b, d, f	[6,19,33,35,45,46,52,54,61,64]
	**139**	Vitetrifolin E	b, d	[6,45,46,64]
	**140**	Vitetrifolin F	b, d	[6,45,46,64]
	**141**	Vitetrifolin H	d	[45]
Abietane	**142**	Ferruginol	d	[58]
	**143**	Abietatrien-3β-ol	b, d	[39,58]
	**144**	5β-Hydro-8,11,13-abietatrien-6α-ol	a	[65]
	**145**	3β-Hydroxyabieta-8,11,13-trien-7-one	a	[37]
	**146**	Isolophanthin A	d	[45]
	**147**	Abieta-9(11),12-diene	d	[66]
	**148**	Vitetrifolin A	b	[39]
	**149**	Abietane 9(11):12(13)-di-α-epoxide	d	[66]
	**150**	Vitexifolin C	d	[61]
	**151**	Negundoin F	a	[37]
Clerodane	**152**	Vitexifolin B	d	[61]
	**153**	Cleroda-7,14-dien-13-ol	f	[41]
	**154**	Cleroda-1,3,14-trien-13-ol	f	[41]
	**155**	13-*epi-*2-Oxokolavelool	d	[45]
Isopimarane	**156**	Negundoin G	a	[37]

a: *Vitex negundo*. b: *V. trifolia*. d: *V. rotundifolia*. f: *V. agnus-castus*. g: *V. limonifolia*. h: *V. negundo* var. *cannabifolia* (syn.: *V. cannabifolia*). j: *V. vestita*. k: *V. rehmannii*. l: *V. trifolia* L. var. *simplicifolia*.

**Table 4:** Triterpenoids **157**–**210** isolated from plants of *Vitex* L.

Type	No.	Compound Name	Source	Reference
Oleanane	**157**	Oleanolic acid	a, b	[54,68,70,71]
	**158**	Maslinic acid	a, c, f	[33,54,72,73]
	**159**	3-Epimaslinic acid	a, c, f, h	[33,51,69,71,73]
	**160**	2α,3α,24-Trihydroxyolean-12-en-28-oic acid	a, b, h	[69,72,74]
	**161**	2α,3β,24-Trihydroxyolean-12-en-28-oic acid	b	[74]
	**162**	Vulgarsaponin A	a	[67]
	**163**	Cannabifolin F	h	[69]
	**164**	Oleanolic acid acetate	a	[75]
	**165**	Hederagenin	b	[68]
	**166**	2α,3α,23-Trihydroxyolean-12-en-28-oic acid methyl ester	a	[71]
	**167**	2α,3α,23-Trihydroxyolean-12-en-28-oic acid	a	[71]
	**168**	2α,3β,19α,23-Tetrahydroxyolean-12-en-28-oic acid	a	[72]
	**169**	2α,3β,23-Trihydroxyolean-12-en-28-oic acid	a	[72]
	**170**	3β-Hydroxyolean-5,12-dien-28-oic acid	a	[76]
	**171**	2α,3α-Dihydroxyoleana-5,12-dien-28-oic acid	a	[77]
	**172**	2β,3α-Diacetoxyoleana-5,12-dien-28-oic acid	a	[77]
	**173**	2α,3β-Diacetoxy-18-hydroxyoleana-5,12-dien-28-oic acid	a	[77]
	**174**	Taraxerol	b	[78]
	**175**	Taraxerone	l	[79]
	**176**	3-Oxotaraxer-14-en-30-al	l	[79]
	**177**	β-Amyrin	a, b	[68,71]
	**178**	β-Amyrin-3-*O*-β-d-glucopyranoside	b	[68]
	**179**	3β-Acetoxyolean-12-en-27-oic acid	a	[76,77]
	**180**	Cannabifolin E	h	[69]
	**181**	23-Hydroxy-3α-[*O*-α*-*l-rhamnopyranosyl-(1′′′→4″)-*O*-[β-d-(*E*-6″-*O*-caffeoyl)-glucopyranosyl]-oxy]-olean-12-en-28-oic acid	b	[68]
	**182**	23-hydroxy-3α-(*O*-sulfonyloxy)-olean-12-en-28-oic acid-28-*O*-[α-l-rhamnopyranosyl-(1′′′→4″)-*O*-β-d-glucopyranosyl-(1″→6′)-*O*-β-d-glucopyranosyl] ester	b	[68]
	**183**	Cannabifolin B	h	[69]
Ursane	**184**	Cannabifolin A	h	[69]
	**185**	Ursolic acid	a–c, e, h	[49,53,54,69,72,73,78,80,81]
	**186**	3-Epiursolic acid	b, l	[74,79]
	**187**	Corosolic acid	a–c, e, f, h, l	[33,53,67,69,73,79,80]
	**188**	3-Epicorosolic acid	a–c, f, h	[28,33,51,69,73,78]
	**189**	3β-Acetoxyurs-12-en-28-oic acid	b	[49,82]
	**190**	α-Amyrin	b	[53]
	**191**	Uvaol	b	[74]
	**192**	Tormentic acid	a, b, e, h	[69,72,78,83]
	**193**	2α,3α,24-Trihydroxyurs-12-en-28-oic acid	b, c	[73,74]
	**194**	Euscaphic acid	c, h	[69,73]
	**195**	2α,3α,24-Trihydroxyurs-12-en-28-oic acid-28-*O*-β-d-glucopyranosyl ester	a	[67]
	**196**	2α,3α,24-Trihydroxyurs-12,20(30)-dien-28-oic acid-28-*O*-β-d-glucopyranosyl ester	a	[67]
	**197**	2α,3α,24-Trihydroxyurs-12,20(30)-dien-28-oic acid	c	[73]
	**198**	2α,3α-Dihydroxyurs-12,20(30)-dien-28-oic acid	h	[69]
	**199**	Cannabifolin C	h	[69]
	**200**	Cannabifolin D	h	[69]
	**201**	Ilelatifol D	f	[51]
Norursane	**202**	Negundonorin A	a	[28]
	**203**	Negundonorin B	a	[28]
Lupane	**204**	Lupeol	l	[79]
	**205**	Betulinic acid	a, b, l	[49,54,78,79,81]
	**206**	Lup-20(29)-en-3β,30-diol	a	[54]
	**207**	Obtusalin	a	[54]
	**208**	Platanic acid	b	[82]
Friedelane	**209**	Epifriedelinol	e	[84]
9-*epi*-Cucurbitane	**210**	(24*R/S*)-24-Hydroxy-3α,10α-epoxy-9-*epi*-cucurbita-25-ene	a	[43]

a: *Vitex negundo*. b: *V. trifolia*. c: *V. altissima*. e: *V. peduncularis*. f: *V. agnus-castus*. h: *V. negundo* var. *cannabifolia* (syn.: *V. cannabifolia*). l: *V. trifolia* L. var. *simplicifolia*.
